# The Role of Low Self-Control as a Mediator between Trauma and Antisociality/Criminality in Youth

**DOI:** 10.3390/ijerph18020567

**Published:** 2021-01-12

**Authors:** Pedro Pechorro, Matt DeLisi, Rui Abrunhosa Gonçalves, João Pedro Oliveira

**Affiliations:** 1 School of Psychology, Campus de Gualtar, University of Minho, 4710-057 Braga, Portugal; rabrunhosa@psi.uminho.pt; 2 Department of Sociology and Criminal Justice, Iowa State University, 203A East Hall, Ames, IA 50011-1070, USA; delisi@iastate.edu; 3 School of Psychology and Life Sciences, Lusófona University of Humanities and Technologies, Campo Grande, 1749-024 Lisbon, Portugal; joaopoliveira@yahoo.com

**Keywords:** aggression, conduct disorder, juvenile delinquency, mediation, self-control, trauma

## Abstract

Trauma exposure and low self-control are robustly associated with youth antisocial/criminal problems, but the interrelation of these constructs is unclear when taking into account both traumatic events and reactions. The objective of the present study is to examine self-control mediation effects related to trauma and juvenile delinquency, conduct disorder, crime seriousness, and aggression outcomes. The sample consisted of *N* = 388 male youth from Portugal (age, *M* = 16.01 years, *SD* = 1.03, age range = 13–18 years). Path analysis procedures revealed that self-control partially mediates the relation between trauma events and the examined outcomes and fully mediates the relation between trauma reactions and the examined outcomes. Research on youth trauma should examine both trauma events and trauma reactions because they have differential effects on low self-control and antisocial/criminal outcomes.

## 1. Introduction

Evidenced by the child maltreatment and cycle of violence literature [1,2,3], social scientists have long recognized the immediate and long-term sequela that various forms of abuse and neglect have on youth development and behavioral functioning. Although the precise mechanisms by which childhood trauma translates into conduct problems are complex [4,5], it is clear that both the original traumatic experience and the subsequent psychological and behavioral reactions to it play a role in antisocial development. Over the past decade, this research enterprise has grown and the study of adverse childhood experiences and various forms of childhood trauma has become a central research area in criminology and related disciplines [6].

Several important findings emanate from the criminological study of traumatic experiences among youth. Childhood trauma has broad, generalized effects whereby adverse experiences are associated with increased conduct problems [7,8,9,10] and localized effects where specific forms of abuse manifest in analogous variants of delinquency, for instance, sexual abuse manifesting as sexual aggression [7,9,11]. Thus, childhood trauma has multifaceted and seemingly idiosyncratic effects on youth development. Various forms of abuse (e.g., physical, sexual, and emotional), neglect (e.g., physical, emotional, medical, and educational), and other traumas (e.g., abandonment, family disruption, exposure to drugs, violence, and crime) affect youth in potentially different ways. As Westermair et al. [12] advised, “all unhappy childhoods are unhappy in their own way”.

Traumatic experiences spanning diverse forms of abuse and neglect tend to cluster [6,10,13], such that youth exposed to one form of trauma are significantly likely to experience multiple traumatic experiences. The cumulative burden of traumatic experiences is costly, with research on nearly 25,000 delinquent youth in the United States indicating that each additional traumatic experience increases the likelihood of serious, violent, and chronic delinquency by 35% [14]. This suggests that youth exposed to the greatest amount of trauma are dramatically more likely to exhibit pathological conduct problems.

On this empirical foundation, recent research has attempted to specify the personality and dispositional characteristics that result from traumatic exposure as well as potentially mediate the association between adverse events and delinquency [15,16,17,18,19]. Prominent in criminology [20], low self-control is a disposition characterized by poor emotional regulation or temper, impulsivity, self-centeredness, action as opposed to cognitive orientation, and poor gratification delay and has broad associations with antisocial behavior [21,22,23,24]. Self-control deficits are theorized to be the outcome of inadequate parental socialization where there is low monitoring and inconsistent and negligent sanctioning of conduct problems [20]. Across a variety of data sources of youth, low self-control appears to be both a consequence of traumatic experiences and one that mediates trauma linkages to delinquency. Employing data from more than 64,000 juvenile delinquents in the United States, for example, the authors of [25] found that between 37% and 93% of the effects of traumatic experiences on subsequent juvenile justice system contacts are mediated by self-regulation deficits inherent to psychopathic personality features.

Several studies have shown that self-control features, such as impulsivity, temper/aggression, non-cognitive orientation, and related psychopathology, are produced by traumatic experiences or mediate associations between traumatic experiences and serious delinquency [26,27]. For instance, Xie et al. [19] studied 585 male juvenile delinquents in China and found that greater childhood maltreatment was associated with self-control deficits. In addition, self-control completely mediated the association between childhood maltreatment and adolescent aggression [19].

Thus, one reason that traumatic experiences increase liability for conduct problems relates to increases in psychopathology, specifically self-control deficits that result from abuse and neglect and mediate their connection to delinquency. Although trauma and self-control are recurrent in antisocial/criminal problems among youth, it is still not clear how these are interrelated. Prior studies have overwhelmingly employed correctional or adjudicated samples of juvenile delinquents, but the broader application to males in the community is sparse. Research needs to examine if trauma events and trauma reactions differentially influence low self-control and if low self-control mediates their associations with antisocial/criminal outcomes. The current study sought to fill this void. To explore this, we examined the following four hypotheses: (i) self-control mediates the relation between trauma and the juvenile delinquency outcome, (ii) self-control mediates the relation between trauma and the Conduct Disorder (CD) outcome, (iii) self-control mediates the relation between trauma and the crime seriousness outcome, and (iv) self-control mediates the relation between trauma and the proactive aggression outcome.

## 2. Materials and Methods

### 2.1. Participants

The sample consisted of *N* = 388 male youth from Portugal (age, *M* = 16.01 years, *SD* = 1.03, age range = 13–18 years). Participants had approximately eight years of education (*M* = 8.14, *SD* = 1.63, range = 4–11 years) and (58.2%) had low socioeconomic status.

### 2.2. Measures

#### 2.2.1. Predictor

*Child Trauma Screen* (CTS; [28,29]). This is a brief, empirically derived measure designed to screen for trauma across child and youth serving systems. The CTS includes four dichotomous trauma exposure (event) items (e.g., Has someone ever really hurt you? Hit, punched, or kicked you really hard with hands, belts, or other objects, or tried to shoot or stab you?), and six ordinal reactions items that assess Post-Traumatic Stress Disorder (PTSD) symptoms (e.g., How often did each of these happen in the last 30 days? Try to stay away from people, places, or things that remind you about something that happened.). The CTS can be scored by adding the items of the reactions factor on a 4-point ordinal scale (ranging from 0 = Never/Rarely, to 3 = Three or more times per week). The trauma exposure (event) items can be added. However, the use of a total score that includes the trauma exposure event items is not recommended. Because of that, no total score was used in the current study, and only the internal consistency of the reaction factor was provided. Higher scores on the reaction factor indicate higher levels of PTSD. The CTS was translated and validated in Portugal among the youth school population [30] and Cronbach’s α = 0.78.

#### 2.2.2. Mediator

*Brief Self-Control Scale* (BSCS; [31]). This is a brief, 13-item, self-reported, unidimensional measure of self-control. The BSCS includes items such as: “I refuse things that are bad for me”; “I am able to work effectively toward long-term goals”; “sometimes I can’t stop myself from doing something”. Items are rated on a 5-point Likert scale (ranging from 1 = Not at all like me to 5 = Very much like me). The total score of the BSCS can be obtained by adding the items. Items were reverse-scored so higher scores reflect lower levels of self-control. The version of the BSCS translated and validated in Portugal among the youth population was used in the current study [32] and Cronbach’s α = 0.94.

#### 2.2.3. Outcomes

*Add Health Self-Report Delinquency* (AHSRD). This is a 17-item, self-reported measure of juvenile delinquency originally developed for the National Longitudinal Study of Adolescent to Adult Health (Add Health). The AHSRD taps criminal behaviors occurring during the last year before the assessment (e.g., taking something from a store without paying for it, stealing something worth less than €50, or buying, selling, or holding stolen property). Items are rated on a 4-point Likert scale (ranging from 0 = None, to 3 = Five or more times). The total delinquency score can be obtained by adding the items. Higher scores indicate higher levels of self-reported criminality. The AHSRD was translated and validated in Portugal among the youth population [33] and Cronbach’s α = 0.96.

*Conduct Disorder Screener* (CDS; [34]). This is a brief, self-reported screener created to identify adolescents with conduct disorder. The CDS consists of six items representative of a diagnosis of conduct disorder (e.g., I broke rules at school; I got in trouble for lying or stealing). The CDS can be scored by adding the items on a 4-point ordinal Likert scale (ranging from 1 = Rarely or none of the time, to 4 = Most or all of the time). Higher scores indicate higher levels of conduct disorder. The CDS was translated and validated in Portugal among the youth forensic population [35] and Cronbach’s α = 0.92.

*Peer Conflict Scale-20* (PCS-20; [36]). This is a brief, 20-item, self-reported, four-dimensional measure that taps the different forms and functions of aggression including proactive overt, reactive overt, proactive relational, and reactive relational aggression. We focused solely on proactive overt aggression because it is the most serious and explicitly direct form of aggression in the measure. The PCS-20 has five proactive overt (PO) items (e.g., I start fights to get what I want) and items are rated on a 4-point Likert scale (ranging from 0 = Not at all true, to 3 = Definitely true). Higher scores indicate higher levels of aggression. The PCS-20 was translated and validated in Portugal among the youth forensic population [37] and Cronbach’s α = 0.91 for PO aggression.

*Delinquency Seriousness Classification Index* (DSCI). A Portuguese version of the index of crime seriousness originally developed by [38] was used to classify crime seriousness. This version employs a four-level progressive ordinal sequence, with higher scores indicating higher seriousness levels of crimes committed by youth (e.g., level 4 consists of serious delinquency such as car theft and breaking and entering).

### 2.3. Procedures

The Portuguese State (Ministério da Educação) provided authorization to assess the participants of the present study. The study was conducted in accordance with the Declaration of Helsinki, and the protocol was approved by the Ethics Committee of the Direção-Geral de Educação (Code: 0618500001). Written parental permission was obtained for all underage children, and an informed consent form was obtained if participants were 18 years of age or older. The participants came from schools and community education centers in Portugal, including the capital city Lisbon, some of which were situated in impoverished and disadvantaged zones characterized by social exclusion, high unemployment rates, and limited economic resources. The potential participants were themselves informed about the aims of our investigation and asked to collaborate voluntarily and anonymously. The first author, who is a professional researcher with a PhD in Forensic Psychology, collected the surveys from participants. The completed surveys were stored at a safe location where only the researchers could have access to them. Due to various motives, some youth were excluded (e.g., those not able to understand Portuguese or those who could not read the survey independently). The measures and sociodemographic questionnaire included in the present study were administered in small groups of participants. The rate of participation was 89%. No monetary compensation or other forms of compensation were given to the participants or their parents.

### 2.4. Data Analysis

The EQS 6.4 software [39] was used to examine descriptive statistics, Pearson’s correlations, and reliability of the measures (i.e., Cronbach’s α) and to estimate path analysis models (that is, to analyze the direct and indirect effects between trauma and the outcomes). Pearson’s correlations were considered to be high if above 0.50, moderate if between 0.20 and 0.50, and low if below 0.20 [40]. The alpha coefficient was considered adequate if above 0.70 and good if above 0.80 [41]. Path analysis using constructs score was used to overcome sample size limitations and estimation errors. When the reliability of the constructs is high, the underestimation of the beta coefficients using path analysis is irrelevant [42]. We tested four fully saturated models. In Model 1, trauma predicted self-reported delinquency directly and indirectly via its influence on self-control. In Model 2, trauma predicted CD directly and indirectly via its influence on self-control. In Model 3, trauma predicted crime seriousness directly and indirectly via its influence on self-control. In Model 4, trauma predicted proactive, overt aggression directly and indirectly via its influence on self-control. We considered that with complete mediation, the entire (or total) effect of an independent variable on a dependent variable is transmitted through the mediator variable, that is, the independent variable has no direct effect on the dependent variable; rather, its entire effect is indirect. On the other hand, with partial mediation, the independent variable has both direct and indirect effects on a dependent variable, that is, the direct effect is not mediated, whereas the indirect effect is transmitted through the mediator variable. No modification indices were used to improve these models. Maximum Likelihood (ML) estimation methods with Pearson’s covariance matrices were used. Those methods are considered robust for non-severe violations of the normality (absolute skewness and kurtosis values below 3 and 7, respectively [42]).

## 3. Results

Table 1 presents the Pearson’s correlation matrix of the measures used. As expected, the correlation between the measures was moderate to high and statistically significant (ranging from 0.26 to 0.92). Traumatic events tended to present stronger correlations with the remaining variables than trauma reactions.

**Figure 1:**
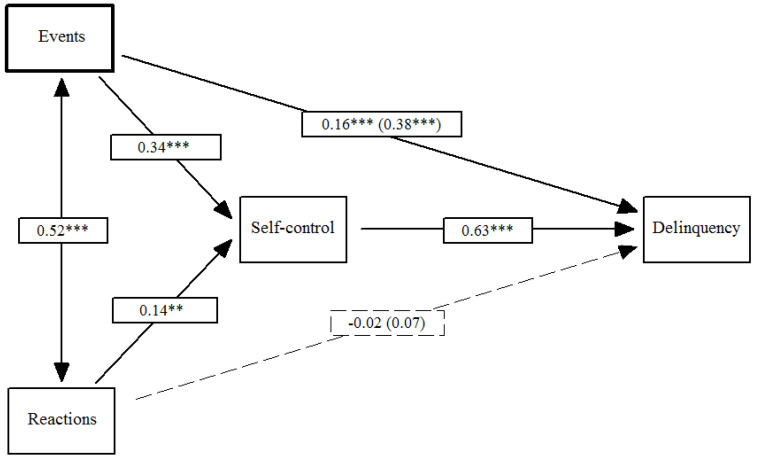
Effects of traumatic events and reactions on self-reported delinquency mediated by low self-control. Note: Total effects are included in parentheses; ** *p* ≤ 0.01, *** *p* ≤ 0.001.

Figure 1 displays the first model. The traumatic events and trauma reactions of the CTS had a positive impact on low self-control (*β*_Events_ = 0.34, *p* ≤ 0.001; *β*_Reactions_ = 0.14, *p* ≤ 0.01), which in turn predicted self-reported delinquency (*β*_Self-control_ = 0.63, *p* ≤ 0.001). The total effect of the events and reactions on self-reported delinquency (*β*_Events_ = 0.38, *p* ≤ 0.001; *β*_Reactions_ = 0.07, *ns*) included both the indirect effect via self-control (*β*_Events_ = 0.21, *p* ≤ 0.001; *β*_Reactions_ = 0.09, *p* ≤ 0.01) and the direct effect on self-reported delinquency (*β*_Events_ = 0.16, *p* ≤ 0.001; *β*_Reactions_ = −0.02, *ns*). In total, the direct and indirect effects of traumatic events on self-reported delinquency were significant. Regarding trauma reactions, only the indirect effect was significant.

**Figure 2:**
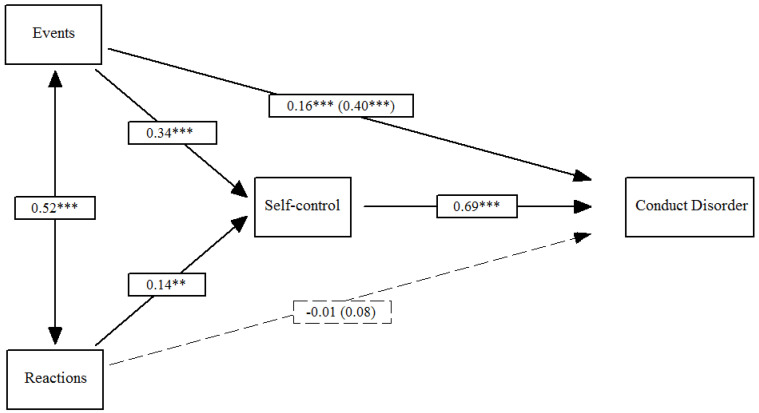
Effects of traumatic events and reactions on conduct disorder mediated by low self-control. Note: Total effects are included in parentheses; ** *p* ≤ 0.01, *** *p* ≤ 0.001.

Next, Figure 2 shows Model 2. Traumatic events and trauma reactions had a positive impact on low self-control (*β*_Events_ = 0.34, *p* ≤ 0.001; *β*_Reactions_ = 0.14, *p* ≤ 0.01), which in turn predicted conduct disorder (*β*_Self-control_ = 0.69, *p* ≤ 0.001). The total effect of the events and reactions on conduct disorder (*β*_Events_ = 0.40, *p* ≤ 0.001; *β*_Reactions_ = 0.08, *ns*) included both the indirect effect via self-control (*β*_Events_ = 0.23, *p* ≤ 0.001; *β*_Reactions_ = 0.10, *p* ≤ 0.01) and the direct effect on conduct disorder (*β*_Events_ = 0.16, *p* ≤ 0.001; *β*_Reactions_ = −0.01, *ns*). In total, the direct and indirect effects of traumatic events on conduct disorder were significant. With regard to trauma reactions, only the indirect effect was significant.

**Figure 3:**
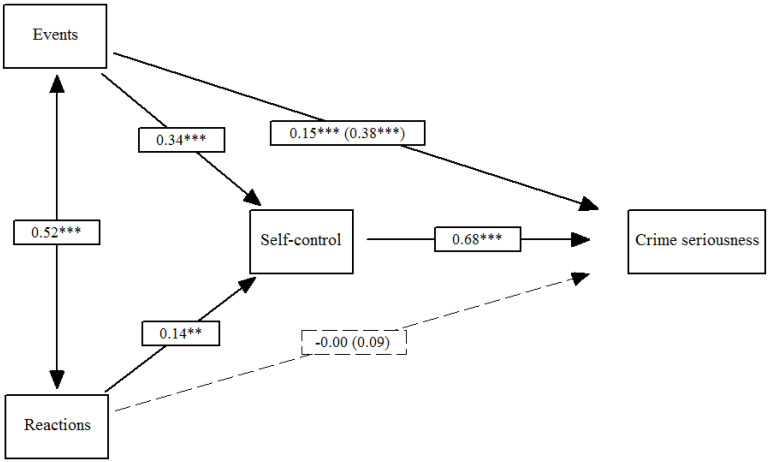
Effects of traumatic events and reactions on crime seriousness mediated by low self-control. Note: Total effects are included in parentheses; ** *p* ≤ 0.01, *** *p* ≤ 0.001.

Figure 3 shows Model 3. Traumatic events and trauma reactions had a positive impact on low self-control (*β*_Events_ = 0.34, *p* ≤ 0.001; *β*_Reactions_ = 0.14, *p* ≤ 0.01), which in turn predicted crime seriousness (*β*_Self-control_ = 0.68, *p* ≤ 0.001). The total effect of the events and reactions on crime seriousness (*β*_Events_ = 0.38, *p* ≤ 0.001; *β*_Reactions_ = 0.09, *ns*) included both the indirect effect via self-control (*β*_Events_ = 0.23, *p* ≤ 0.001; *β*_Reactions_ = 0.10, *p* ≤ 0.01) and the direct effect on crime seriousness (*β*_Events_ = 0.15, *p* ≤ 0.001; *β*_Reactions_ = −0.00, *ns*). In total, the direct and indirect effects of trauma events on crime seriousness were significant. Regarding trauma reactions, only the indirect effect was significant.

**Figure 4:**
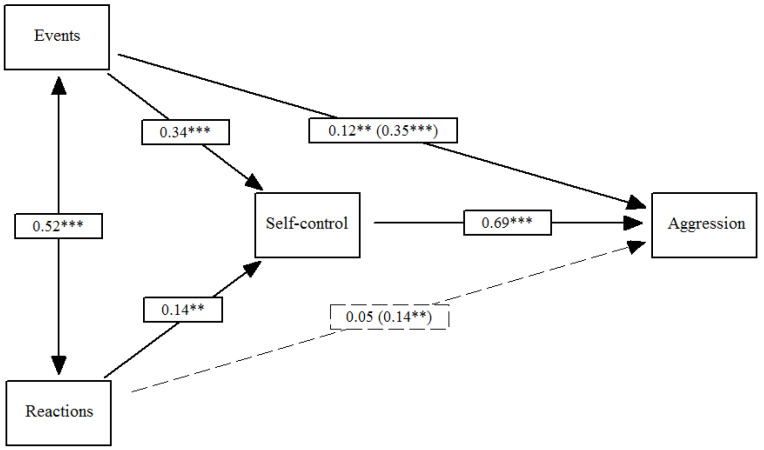
Effects of traumatic events and reactions on proactive, overt aggression mediated by low self-control. Note: Total effects are included in parentheses; ** *p* ≤ 0.01, *** *p* ≤ 0.001.

Finally, Figure 4 shows Model 4. Traumatic events and trauma reactions had a positive impact on low self-control (*β*_Events_ = 0.34, *p* ≤ 0.001; *β*_Reactions_ = 0.14, *p* ≤ 0.01), which in turn predicted proactive, overt aggression (*β*_Self-control_ = 0.69, *p* ≤ 0.001). The total effect of the events and reactions on proactive, overt aggression (*β*_Events_ = 0.35, *p* ≤ 0.001; *β*_Reactions_ = 0.14, *p* ≤ 0.01) included both the indirect effect via self-control (*β*_Events_ = 0.23, *p* ≤ 0.001; *β*_Reactions_ = 0.10, *p* ≤ 0.01) and the direct effect on proactive, overt aggression (*β*_Events_ = 0.23, *p* ≤ 0.01; *β*_Reactions_ = 0.10, *p* ≤ 0.01). In total, the direct and indirect effects of traumatic events on overt aggression were significant. With regard to trauma reactions, only the indirect effect was significant. It is important to mention that the effects reported in these four models take into consideration that traumatic events, and trauma reactions are positively and significantly correlated (*β* = 0.52, *p* ≤ 0.001).

## 4. Discussion

In the current study, we examined traumatic events and reaction effects on antisocial/criminal outcomes and the role of low self-control as a mediator between trauma and those antisocial outcomes in youth. Despite the fact that trauma and self-control are central to the study of antisocial and criminal trajectories among youth [6,16,17,22], the nature of their relationship remains unclear. There is a gap in the literature in terms of simultaneously examining the effects of traumatic events and trauma reactions. Our overall findings indicate that traumatic events and trauma reactions directly, positively predict low self-control, but only traumatic events directly, positively predict the examined outcomes including juvenile delinquency, conduct disorder, crime seriousness, and proactive overt aggression outcomes. The findings also indicate that low self-control partly mediates the relationship between traumatic events and the outcomes, and fully mediates the relationship between trauma reactions and the outcomes. This discussion focuses on the theoretical issues surrounding the etiology of low self-control and a trauma-informed care approach to responding to various forms of abuse and neglect in the community.

In the most influential criminological perspective on self-control [20], it was theorized that “poor parenting”, or ineffective parental socialization characterized by low monitoring, poor recognition of child misbehavior, and erratic, inconsistent, or nonexistent sanctioning of child misbehavior, is what produced low self-control. Indeed, Gottfredson and Hirschi discussed a variety of family and child-rearing factors including parent–child attachment, parental supervision, parental criminality, family size, family structure, and mothers working outside the home [20]. These are normative family issues and theoretically, deficits in one or more areas inculcate less than optimal self-control in children.

Consistent with other recent studies [17,18,25,26,27], the current models show convincingly that the etiology of low self-control also has a more pathological basis in terms of trauma exposure, a concept largely overlooked in [20]. Moreover, trauma has a double-pronged effect on self-control as the abusive, neglectful, or traumatic event and the emotional and psychological reaction to it reduce self-control in youth. Traumatic events and trauma reactions are strongly related, but they have different effects on low self-control, which in turn differently mediates their relationship with antisocial/criminal outcomes. The original traumatic experiences and the subsequent reactions to the traumatic experiences seem to play a role that is not identical in terms of causing antisocial/criminal development. For example, the authors of [4] did not find sufficient evidence that adverse childhood experiences have a direct effect on antisocial behavior and suggested that unmeasured factors (e.g., familial or other) related to adverse childhood experiences may be responsible for higher levels of antisocial behaviors and violent victimization. Thus, there is a need for researchers to identify alternative, plausible causal risk factors associated with trauma/adverse childhood experiences to provide more effective targets for interventions.

Given the role of trauma in the etiology of self-control, there is also room for the extension of self-control theories to include both normative parental socialization factors as well as more pathological parental socialization that involves the infliction of various forms of abuse and neglect, as seen in the coercive parenting literature [43,44,45]. For instance, analyses of adult correctional clients at midlife found that offenders who were introduced to substance use early in life, often in the form of direct modeling and the introduction of the substance, had significant self-regulation deficits across their life course and recurrent involvement in the juvenile and criminal justice systems [46]. In terms of extending the self-control theory, it is also potentially fruitful to disaggregate adverse experiences or different forms of trauma to assess their potentially differential linkages to self-control. A recent study of a school-based sample from the Supporting Healthy Adolescent Relationships and Environments (SHARE) study and a juvenile justice sample from the Florida Department of Juvenile Justice found that although traumatic experiences are generally associated with self-control deficits, some experiences are more salient than others [17]. Specifically, interpersonal maltreatment was more robustly associated with self-control deficits than household dysfunction. Thus, the current findings provide avenues for additional research on the parenting etiological factors and the role of specific trauma forms in self-control.

Consistent with prior research using both community [47,48,49] and adjudicated [50,51,52,53] samples of youth, low self-control was robustly associated with multiple forms of externalizing conduct spanning delinquency, conduct disorder, serious crime, and proactive overt aggression, which supports the generality of the construct. The recurrent linkage of self-control deficits to conduct problems makes clear its importance as a target for behavioral interventions. The fortunate news is that interventions have demonstrable efficacy at improving parenting practices [54] that serve to inculcate self-control in children [55]. However, it is also important to recognize the contextual features that appear to engender self-control deficits in youth, especially if those deficits are borne from exposure to traumatic events and psychological problems related to them. Given that both traumatic events and reactions were significantly associated with low self-control, it seems likely that self-control problems are more reactive in response to their trauma. For example, in the event that the traumatic event reflected an act of betrayal, such as physical neglect, medical neglect, physical abuse, or sexual abuse by a parent or guardian, self-regulation problems could be a means by which the youth attempts to hold the abusive or neglectful parent responsible for their conduct and exact a sort of “revenge” against them. This has a very different valence than the proactive overt aggression and highly externalizing valence that is seen in self-control deficits among adjudicated youth. A holistic understanding of the likely causes of the self-control problems can assist counselors and treatment staff with understanding why the youth is displaying self-control problems as well as with providing access to trained practitioners in areas of social service need (e.g., sexual assault survivor treatment).

## 5. Conclusions

In closing, we acknowledge two key limitations of the study that, hopefully, future research can surmount. First, the cross-sectional design precludes the causal ordering of variables; thus, longitudinal designs are necessary to empirically examine the directionality and possible reciprocal associations between trauma and self-control. Second, our study is partially limited by shared method variance as all data are derived from self-reports. Official records of traumatic experiences, as well as additional data on parenting experiences that are germane to the inculcation of self-control, would have been helpful to corroborate the self-reports herein, which is useful since youth have a motivation to both minimize or exaggerate their early-life experiences. Additionally, official psychiatric information would help to inform understanding of PTSD symptoms as well as model additional internalizing and externalizing conditions that could result from trauma exposure.

## Figures and Tables

**Table 1:** Correlation matrix.

	CTS Events	CTS Reactions	BSCS	AHSRD	CDS	DSCI	PCS-20 PO
CTS Events	-						
CTS Reactions	0.52 ***	-					
BSCS	0.41 ***	0.32 ***	-				
AHSRD	0.42 ***	0.26 ***	0.69 ***	-			
CDS	0.44 ***	0.29 ***	0.75 ***	0.92 ***	-		
DSCI	0.43 ***	0.29 ***	0.74 ***	0.88 ***	0.90 ***	-	
PCS-20 PO	0.42 ***	0.32 ***	0.75 ***	0.88 ***	0.88 ***	0.84 ***	-
*M* (*SD*)	1.70 (0.95)	6.97 (4.01)	35.80 (8.44)	12.40 (13.51)	11.86(4.45)	2.29 (1.95)	3.98 (4.08)

Note: CTS = Child Trauma Screen; BSCS = Brief Self-Control Scale; AHSRD = Add Health Self-Report Delinquency; CDS = Conduct Disorder Scale; DSCI = Delinquency Seriousness Classification Index; PCS-20 PO = Brief Peer Conflict Scale Proactive Overt; *M* (*SD*) = Mean (Standard Deviation). *** *p* ≤ 0.001.

## Data Availability

Data are available upon reasonable request to the first author.
